# Equal quality of life after limb-sparing or ablative surgery for lower extremity sarcomas

**DOI:** 10.1038/sj.bjc.6602104

**Published:** 2004-08-03

**Authors:** A Zahlten-Hinguranage, L Bernd, V Ewerbeck, D Sabo

**Affiliations:** 1 Department of Orthopaedic Surgery, University of Heidelberg, Germany

**Keywords:** lower extremity sarcomas, quality of life, limb-sparing surgery, amputation

## Abstract

This study investigated quality of life (QoL) and function of 124 patients with lower extremity sarcoma who underwent either amputation or limb-salvage surgery (LSS) in order to assess potential differences in subjective treatment outcome. The results reflect similar QoL in both treatment groups. However, in contrast to patients with LSS, who described QoL in terms of a high physical performance status with sports and recreational activities, amputees' QoL was strongly associated with their social acceptability. High QoL in amputees brings into question the expectations held with time-consuming advanced technical skills for LSS by physicians.

For decades, limb-salvage surgery (LSS), time-consuming surgical reconstruction techniques, for lower extremity bone tumours has been, compared to amputation, often associated with better quality of life (QoL) owing to its less disfiguring results and a better potential functional outcome. Little is known about QoL among orthopaedic cancer patients and several authors who raised this issue in clinical trials, in respect of the treatment of sarcoma using ablative surgery or LSS, were unable to find distinct QoL advantages related to this type of surgical procedure (Nagarajan *et al*, 2002). This qualitative study extends previous QoL research in orthopaedic oncology by analysing QoL data separately for functional, psychological, social, and health factors. Our primary objective was to investigate which QoL aspects best discriminate the focus groups. It constitutes a major scientific effort to contribute to the understanding of QoL and to determine which aspects are best predictors of whether QoL is high for amputees or patients with LSS.

## PATIENTS AND METHODS

In reviewing the authors' institutional computerised cancer registry, we identified 189 eligible survivors of a malignant lower extremity sarcoma who underwent either amputation or LSS during the period 1980–2000. A minimum time interval of 1 year after treatment was defined as inclusion criteria in order to avoid the influence of side effects of adjuvant chemotherapy on QoL. Patients with evidence of tumour progression, tumour site below the upper ankle joint, age below 14 years, or lack of basic proficiency in the German language were excluded from the study.

As 12 patients refused to participate due to either intimate questioning or lack of motivation, and 49 patients were discharged from the care of the hospital prior to the beginning of the study, we provided the data of 124 patients (78 male, 46 female).

A total of 102 patients (82%) had some form of LSS and 22 patients (18%) had an amputation. Table 1

**Table 1:** Clinical characteristics of the study population

	**Limb-sparing median (min–max)/frequency**	**Amputation median (min–max)/frequency**	
	**(*n*=102)**	**(*n*=22)**	**Missing**
Age (at follow-up)	33 (14–75 years)	37 (15–76 years)	0
*Gender*			
Male	63 (62%)	15 (68%)	
Female	39 (38%)	7 (32%)	0
			
*Tumour site*			
Lower leg	8 (8%)	1 (5 %)	
Knee	55 (54%)	(32%)	
Upper leg	24 (24%)	10 (45%)	0
Pelvis	15 (15%)	4 (18%)	
			
*Surgical therapy*			
Limb-sparing	102	—	
Amputation	—	22	0
			
*Limb-salvage surgery (LSS)*			
None	23	—	
Allo-/autograft	34	—	
Endoprosthesis	38	—	
Rotationplasty	7	—	0
			
Interval period (months)			
(Surgery/follow-up)	46 (6–250)	45 (6–216)	0

outlines the clinical characteristics of the study population.

### Measures

Disease-specific QoL was evaluated with the EORTC QLQ-C30 core instrument in its standard German version 3.0. (Fayers *et al*, 2001). It incorporates five functional scales (physical, behavioural (role), emotional, cognitive, and social), three somatic scales, and a global health and QoL scale. Additionally, several single-item symptom measures are included. The items are rated on a four-point Likert scale. All subscale scores are transformed to 0–100 scales, with higher scores on functional scales representing better functioning and higher scores on symptom scales gear symptoms.

Functional evaluation was performed using the Musculoskeletal Tumour Society Score (MSTS) described by Enneking *et al*. (1993). This score is based on six categories – pain, level of activity and restriction, emotional acceptance, use of orthopaedic supports, walking ability, and gait – with a maximum of five points for each category. The sum-score is transformed to a percentage scale. Although the validity and responsiveness of the MSTS-93 to evaluate amputations has not been established, as the value for ‘supports’ is determined by the type and frequency of external supports, its use is accepted for comparative outcome studies of this focus group (van Dam *et al*, 2001).

As generic QoL assessment tool, the Life Satisfaction Questionnaire (FLZ) was used (Fahrenberg *et al*, 2000). It is a 70-item instrument that covers 10 domains: health, employment, finances, recreation, partnership, peer relations, self-perception, sexuality, social relations, and housing. Each scale comprises seven items to be ranged on a 1–7 point Likert scale from ‘highly dissatisfied’ to ‘highly satisfied’. For the evaluation of overall life satisfaction, all subscales except ‘employment’, ‘partnership’, and ‘peer relationship’ are summed up.

Demographic, clinical, and oncological data were retrieved from the cancer registry. All psychometric tests were applied in the same order to the patients and surveyed in interviews conducted by an experienced person not otherwise involved in the patients' medical treatment. The treating physician evaluated functional outcome within the context of follow-up.

### Statistical methods

Descriptive statistics were performed for all variables. Results are shown as mean (*M*)±standard deviation (s.d.). For the profiling of group differences, canonical discriminant analysis was used. Analyses were calculated with figures for function, life domains, and symptoms, as well as their functional ability and QoL as outcome variable. Results were given as the total canonical structure correlation between discriminating variables and discriminant function. The association between dependent and independent variables are expressed as total canonical structure coefficient (csc) of CAN1. All calculations were performed using SAS for Windows Version 8.12 (Statistical Analysis System, Cary, NC, USA).

## RESULTS

**Figure 1:**
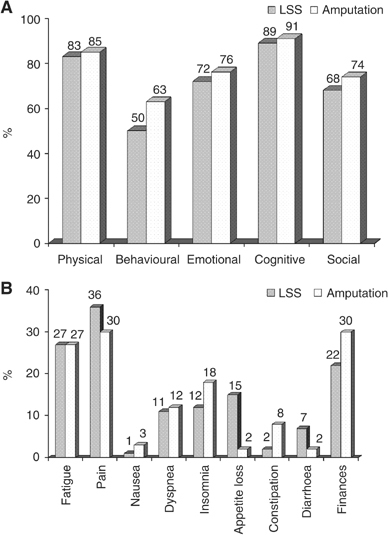
Descriptive statistics for the EORTC QLQ-C30. (**A**) Functional scale, (**B**) Symptom scale.

**Figure 2:**
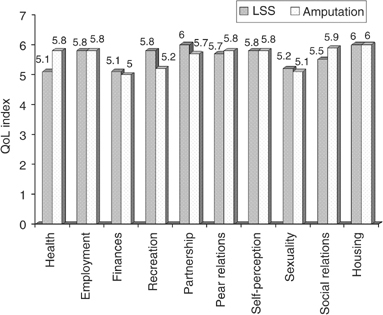
Descriptive statistics for the Life satisfaction Questionnaire (FLZ). QoL-Index.

**Figure 3:**
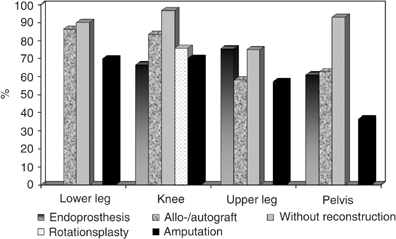
Function (MSTS scores) after LSS and ablative surgery.

Measures of disease-specific as well as global QoL reflect similar results of QoL after LSS or amputation. On a percentage scale, patients with LSS had a mean score of 67.9 (±22.6) and with amputation 68.6 (±22.2), respectively. The overall function scores were slightly higher for those treated with ablative surgery (74.0 (±19.9) *vs* 77.7 (±17.3)). The symptom index was equal with 12.3 (±11.4) *vs* 13.4 (±13.5), respectively (Figure 1

). Global QoL assessment revealed a mean score of 268.8 (±33.4) out of a maximum of 343 points for amputees *vs* 263.8 (±40.1) for patients after LSS, which corresponds to ‘rather satisfied’ on the underlying 1–7 point Likert scale (Figure 2

). The overall functional status was scored with a mean of 21±7 out of 30 points (70%). Descriptive data did indicate a better physical performance status after LSS compared to amputation. Figure 3

provides a visual concept of how function decreased with an increasing anatomical level of surgery.

Discriminant analysis identified physical function with regard to unrestricted level of activity (csc=0.45), independence of assistant device (csc=0.48), unlimited walking ability (csc=0.31), normal gait pattern (csc=0.52), emotional acceptance (csc=0.34), followed by favourable economic conditions (csc=0.23), and recreational activity (csc=0.10) as the most discriminating items with a *P*-value less than 0.05 in the LSS group. The chance probability results in an 85% accurate classification rate. The abilities of physical and cognitive functioning (csc=0.20 and csc=0.14, respectively) enjoyment of good health (csc=0.18), the maintaining of social and sexual relationships (csc=0.10) as well as a low level of pain (csc=0.13) have been found associated with a positive QoL perception in amputees at 73% accurate classification rate.

## DISCUSSION

Our results indicate that there is no benefit of LSS compared to amputation when looking at overall QoL. At an average of 5 years after surgery, both amputees and patients treated with LSS reported an acceptable functional outcome and similar QoL. However, in contrast to patients with LSS, who described QoL in terms of a high physical performance status, amputees' QoL was strongly associated with their social acceptability and good self-reported health.

These findings emphasise the importance of considering social and psychological aspects in QoL research. Particularly, our results demonstrate the link between the integrity of the physical body and self-identity. To question one's social acceptability in terms of avoiding social and sexual relationships is often a consequence of body image concerns and social rejection. Along with others, we support the idea that, due to their visible mutilation, amputees put greater emphasis on interpersonal functioning, such as their social network and emotional or physical intimacies in defining QoL, in order to express their social acceptance (Postma *et al*, 1992; Stevens *et al*, 1996). Given the relationship of health status, usually defined as functional capacity or physiologic functioning and QoL found by van der Schans *et al* (2002), the revealed positive predictive value of improved health, cognitive as well as physical functioning and a low level of phantom pain in amputees was not surprising.

For patients with LSS, those QoL aspects attributed to the physical performance and functional ability were crucial in defining QoL. We suggest that lively recreational and social participation promote a healthy self-image for patients with any limb deficiencies. Precisely because the disability is not apparent, it seems that they try to restore their ‘normality’ by giving themselves a healthy self-image through high recreational and social activity requiring elevated functional capacity. These findings were supported by others, promoting a long-term enhancement of self-esteem through physical activity (Legro *et al*, 2001), underlying the assumption that participation in sports and recreational activity gives a healthy self-image.

As for the functional outcome, our data confirmed improved function after LSS when comparing the MSTS-based results of overall functional outcome with amputees. However, based on the responsiveness of the MSTS for amputees, it should be noted that this advantage is rather a computed value than true superiority in physical functioning, and substantial differences in subjective evaluation are yet to be shown.

In conclusion, the high expectations in terms of QoL put in time-consuming, advanced technical skills for LSS by patients and physicians could not be confirmed in this study. Aside from oncological criteria, the decision to perform LSS should be tempered by the realisation that doing so may lead to repeated hospitalisations, complications, and sometimes a poor functional outcome. Many factors must be considered, including the objective assessment of the patient's physical condition and subjective evaluations related to the patient's psychological, social, and economic status when deciding if a patient will benefit better from early amputation or LSS.
